# Nanosized Water Channels Associated with Hydrophobic and Hydrophilic Fibrillar Arrangements Formed on Nafion Surfaces in Confined Regions

**DOI:** 10.1021/acsomega.4c00809

**Published:** 2024-05-17

**Authors:** Omar Teschke, Paula Simoes Casagrande, David Mendez Soares, Wyllerson Evaristo Gomes

**Affiliations:** † Laboratorio de Nanoestruturas e Interfaces, Instituto de Fisica Gleb Wataghin, UNICAMP, Campinas, 13083-859 São Paulo, Brazil; ‡ Faculdade de Quimica, Pontificia Universidade Catolica de Campinas, Campinas, 13012-970 São Paulo, Brazil

## Abstract

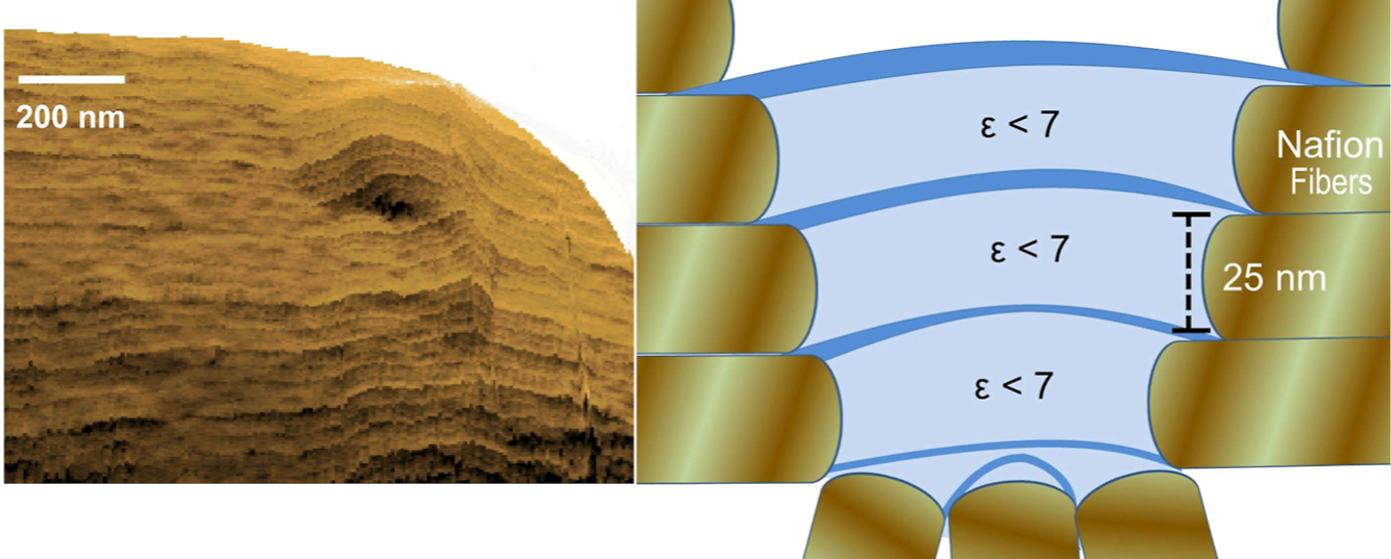

Herein, the origin of interfacial water nanosized channel distributions attached onto Nafion surfaces is investigated. The surface fibrillary hydrophilic and hydrophobic arrangements were observed on AFM images scanned on Nafion surfaces immersed in water. Then, by analyzing the force vs separation curves, it is possible to map arrays of interfacial water channels and their locations. Nafion surface profiles and the water interfacial patterns are then combined using this AFM technique. As there are no reported experimental techniques to measure water nanochannel cross sections, presented measurements report on their dimensions. Water nanochannels characterized by ε < 7 attached to hydrophilic fibrillary sections form aggregated water domains, a highly organized water structure compared with bulk water. Channels are attached to Nafion surface hydrophilic fibrillary domains in confined sites.

## 1 Introduction

Polymer electrolyte fuel cells as a source of clean and sustainable energy conversion devices operating at high current density, moderate operating temperature, and low environmental damage have been investigated and tested.^1,2^ Of the polyelectrolytes, Nafion is one of the most representative ones due to its remarkable proton conductivity and durability.^3−5^ Thus, to improve device performance, it is important to gain a better understanding of the proton transport phenomena not only in the Nafion bulk but also at the Nafion interfaces.

Perfluorosulfonate cation exchange membranes are then used in chlor-alkali electrolyzers and fuel-cell applications because of their high ionic conductivity and their high mechanical, thermal, and chemical stability. Structurally, Nafion consists of a hydrophobic tetrafluoroethylene (TFE) backbone with pendant side chains of perfluorinated vinyl ethers terminated by ion-exchange groups.

Perfluorosulfonate polymers have an ordered structure of hydrophilic end groups within a hydrophobic matrix composed of the fluorocarbon backbone of the polymer. The density contrast between the ionic aggregates and the matrix gives rise to scattering of neutrons^6^ and X-rays.^7,8^ There have been a number of electron microscopy studies of Nafion^9,10^ which support the cluster model of phase separation, although the size scale of the structures observed did not correspond directly with those detected by scattering studies.^6,11,12^ It is important to note that the Nafion membranes in our experiment are highly hydrated and this is reflected in the large size of the hydrophilic domains^13^ as previously reported in our work.^14^

In a recent work,^15^ we reported on interfacial water structures that have been characterized by their dielectric permittivity (ε) profiles and showed that at hydrophilic substrates, there is only one region attached to the substrate where ε is smaller than 7, while hydrophobic substrates show arrangements with variable ε. Anomalously low dielectric constants of ordered interfacial water have been recently reported.^16^ In this work, we investigated the interfacial water dielectric profiles attached to substrates with a mixture of hydrophobic and hydrophilic sites, as the arrangements formed in Nafion surfaces. Nafion was previously used as a separating membrane in pH differential electrolysis cells.^17,18^

In a more recent work,^14^ the immersed Nafion surface fibrillary structure was imaged. The absorbed water in Nafion is separated into domains of the hydrophilic and hydrophobic phases. Ion conductivity occurs through hydrophilic channels.^19,20^ Characterization of the structure and physical properties of Nafion membranes was focused on the structure of hydrophilic domains.^7,21,22^ Small-angle neutron scattering studies and small-angle X-ray studies yielded a new model comprising fibrillary aggregates of a hydrophobic polymer with hydrophilic side chains that protrude radially outward.^19,23,24^ Here, we have, by analyzing the fibrillar arrangement cross section present at the Nafion surface, determined the water structure attached to these surface arrangements.

Water dielectric profiles attached on hydrophobic and hydrophilic sites, forming the Nafion surface structure, were then determined. The dielectric exchange force expression and atomic force microscopy (AFM) force measurement vs separation curves were used to probe and characterize these profiles.

## 2 Experimental Analyses

Swollen membranes were prepared by increasing the temperature from 25 to 100 °C in a vessel for 8 h using pure water as the swelling agent. Four pieces of the membrane were prepared. Pieces of the membrane were weighed in the dry state. After the swelling process, the swollen state of the membrane was determined; all of the samples showed typically a 20% increase in weight. Subsequently, the membrane was introduced in a measuring liquid cell, and force vs distance curves were registered. AFM force measurements were acquired by using a scanning probe microscope (model TMX2000, TopoMetrix, Veeco, Plainview, New York). Silicon nitride (Si_3_N_4_) tips (Veeco, model MSCT-AUHW) with a spring constant of 0.03 N/m and a radius curvature of ∼5 nm were used. The commercial Si_3_N_4_ tip surface has been found to be close to being electrically neutral over a wide pH range (from at least pH 6 to pH 8.5).^25,26^

**Figure 1:**
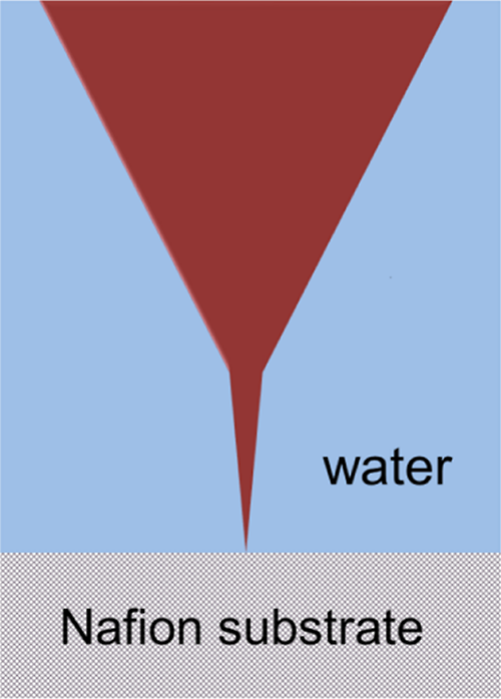
Schematic diagram of the tip/Nafion water interfacial region.

Experiments were performed at room temperature (25 °C) in an environmental chamber housing the AFM. The special feature of our instrument is the liquid cell.^27^ The results reported herein are based on several experiments using different Nafion samples and contact points. A schematic of the Nafion/water interface and probing tip is shown in Figure 1. In order to clarify the environment where the arrangement was formed, we have AFM-scanned Nafion surfaces immersed in water, scanned its topography with a nanosized resolution, and determined the environment where attached to the Nafion surface nanosized water nanochannels are formed.

## 3 Results

Interfacial water is considered to be inhomogeneous because polarization (and permittivity) is a function of the distance to the surface. Variable interfacial profiles were probed using AFM. The measured profiles are induced by the interfacial charged surface.^28−30^ Hydrophobic and hydrophilic sections adjacent to the Nafion substrates immersed in water were probed.

### 3.1 Force vs Separation Profiles Measured at Hydrophobic and Hydrophilic Substrates

**Figure 2:**
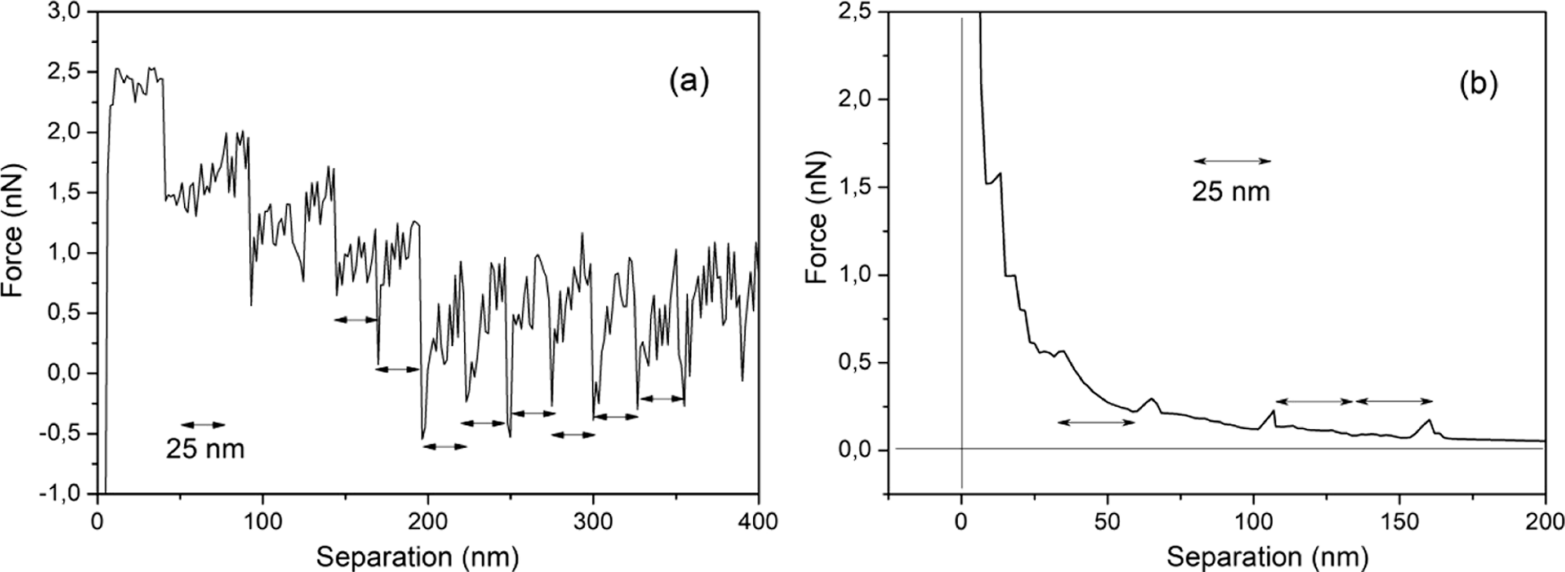
(a) Water/Nafion interfacial region-measured force vs separation curve and (b) another probing region. The values indicated by arrows correspond to repetitive ∼25 nm sections.

A Nafion surface immersed in water force vs separation curve is depicted in Figure 2a. The presented profile can be interpreted as follows. Zero force is recorded beyond ∼400 nm because the AFM tip experiences negligible resistance moving through the bulk as it approaches the Nafion surface. At ∼400 nm, repulsive force, followed by attractive forces, acts on the tip forming a stepped profile with a periodicity of ∼25 nm as indicated by horizontal arrows. The magnitude of these forces for each step varies as the tip moves closer to the surface. Eight repulsive/attractive steps in the force vs separation curves are shown. These profiles characterize the channel structure in the interfacial region that will be discussed in the Discussion section. Figure 2b shows the force vs separation curve for a Nafion/water interfacial region at a distinct location, and the same step structure is observed but with a smaller number of steps. The ∼25 nm or 2 × 25 nm separations are observed, but in Figure 2b, the 25 nm sections show a repulsive force component, while in Figure 2a, the 25 nm sections are attractive.

Figure 2a depicts a strong attraction at the origin (*x* = 0) which is followed for larger distances from the surface by a repulsive component that decreases with the distance from the surface. Observe that this strong attraction at *x* = 0 is not observed in the water profile shown in Figure 2b. So, two distinct patterns of force vs separation curves were registered along the interfacial region.

In order to characterize hydrophobic/hydrophilic sections attached to the Nafion surface, two distinct force profiles of hydrophobic and hydrophilic surfaces published in our previous work^14^ are compared to Figure 2a,b. Mica surfaces immersed in water show the characteristic force vs separation profile where repulsion is observed up to ∼10 nm away from the surface followed by an attractive region where the permittivity is lower than ε_tip_ ∼ 7.^15^ This pattern is shown in Figure 2a.

Hydrophobic surfaces display force vs separation profiles characterized by a strong repulsive force on the tip when immersed in the region attached to the surface, as shown in Figure 2b. Nafion surfaces immersed in water then present regions with hydrophilic and hydrophobic responses when probed by AFM which are shown by the repulsive (hydrophobic) and attractive (hydrophilic) regions attached to their surfaces in force vs separation profiles. Nafion surface hydrophilic/hydrophobic section distribution will be characterized by AFM surface imaging in water as follows.

### 3.2 Hydrated Nafion Surface Morphology and Topography as Observed by AFM

**Figure 3:**
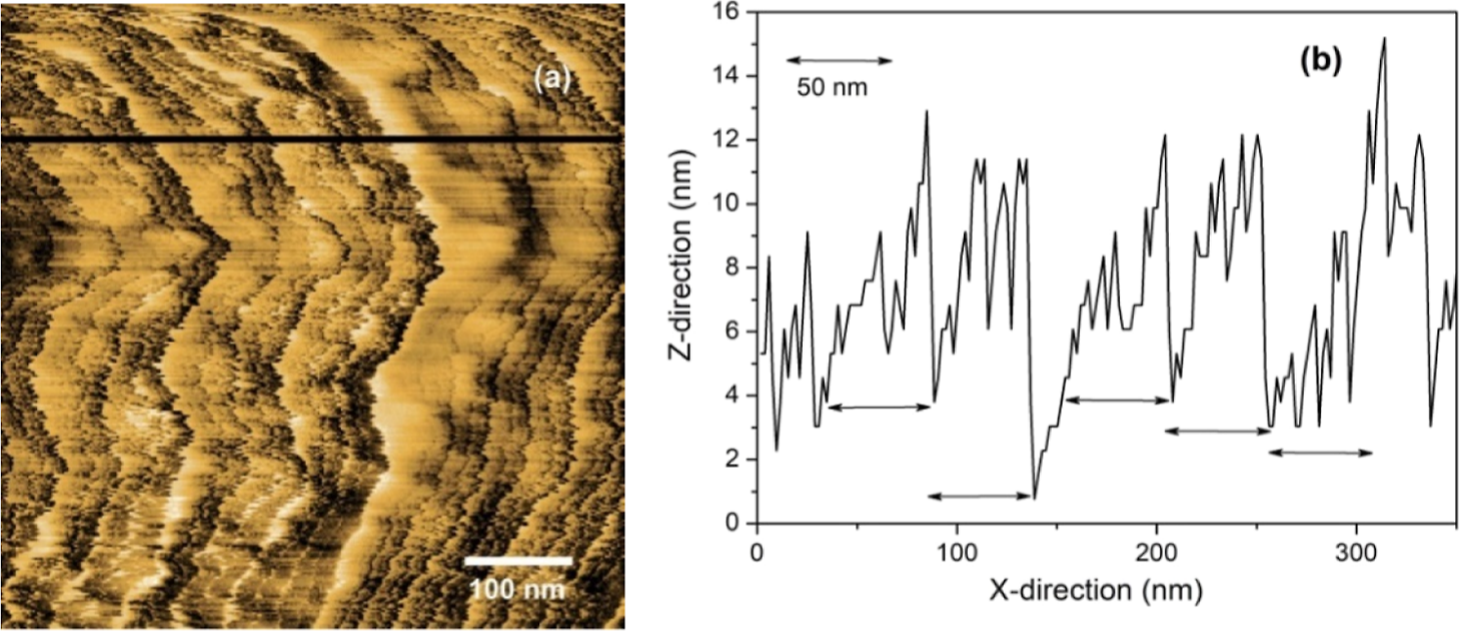
(a) Nafion surface view and (b) surface profile indicated by the horizontal line in (a) only; the first half of the indicated line is displayed. Observe a visible ∼50 nm periodicity indicated by horizontal arrows which corresponds to fiber pairs (2 × 25 nm). This periodicity is associated with the fiber transverse dimension (50 nm) and corresponds to fiber pair widths visible in (a). Adapted from *ACS Omega***2023,***8,* 51, and 49073–49079.

In order to determine the Nafion surface nanosized structure, we have imaged its surface to characterize its nanoscale and macroscopic profiles. Figure 3a shows an image of probed regions, where a macroscopic undulated structure is depicted. This image was registered using Nafion reinforced by a PTFE mesh which has an undulated topography associated with the PTFE wires covered by a Nafion over layer.

Figure 3b shows the profile of an arrangement displayed in Figure 3a (50 nm diameter). The 50 nm corresponds to the diameter of pairs of fibers with 25 nm width. Hydrophilic sites attached to Nafion substrates protrude radially outward,^19,20,23,24^ as depicted in Figure 3a by the light regions.

**Figure 4:**
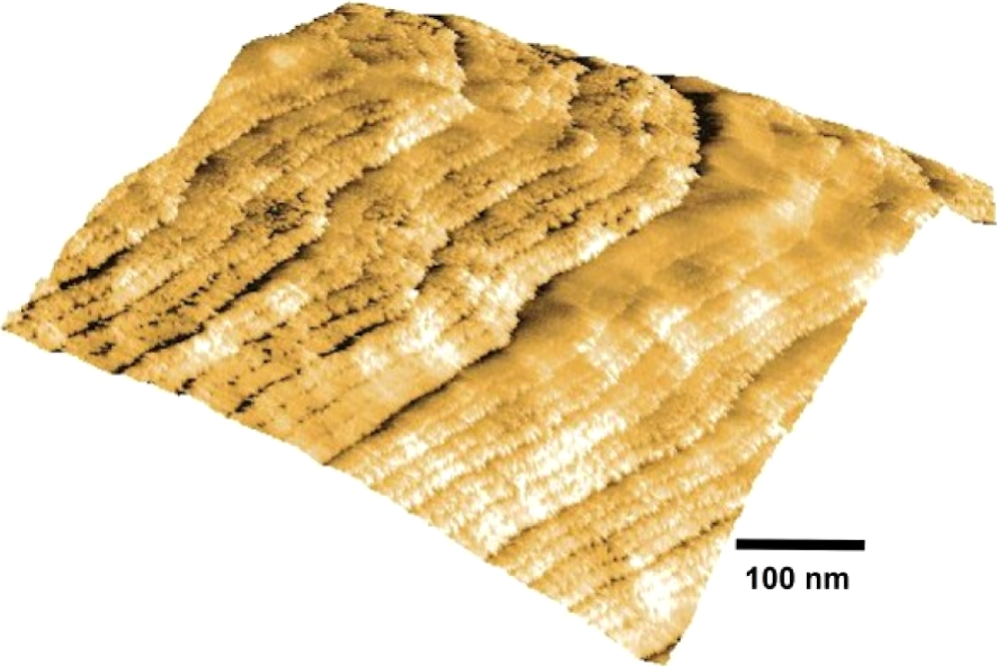
Tridimensional topographic view of a trough is shown in Figure 5a. The depth is ∼175 nm, and the extension is 600 nm along the *X* and *Y* axes.

In order to characterize the surface topography where the water arrangement is observed, tridimensional views were registered. A partial tridimensional view of a protrusion with a depth of typically 175 nm and an extension of 600 nm is shown in Figure 4. A pattern of fibers that are almost aligned horizontally is visible. The figure also shows regions with a highly curved arrangement of fibers attached to a variable surface curvature. Dark regions correspond to lower structures. The Nafion surface morphology is then described as a network of elongated domains that stretch up in length, confirming predictions of large features made by Kim et al.^31^ based on ultrasmall-angle neutron scattering. Hydrated Nafion surfaces formed by an arrangement of hydrated sulfonic acid groups surrounded by a hydrophobic substrate forming fibrillary features were previously shown.^20^ Sites attached to hydrated Nafion substrates then protrude radially outward,^19,23,24^ as observed in Figures 3 and 4.

**Figure 5:**
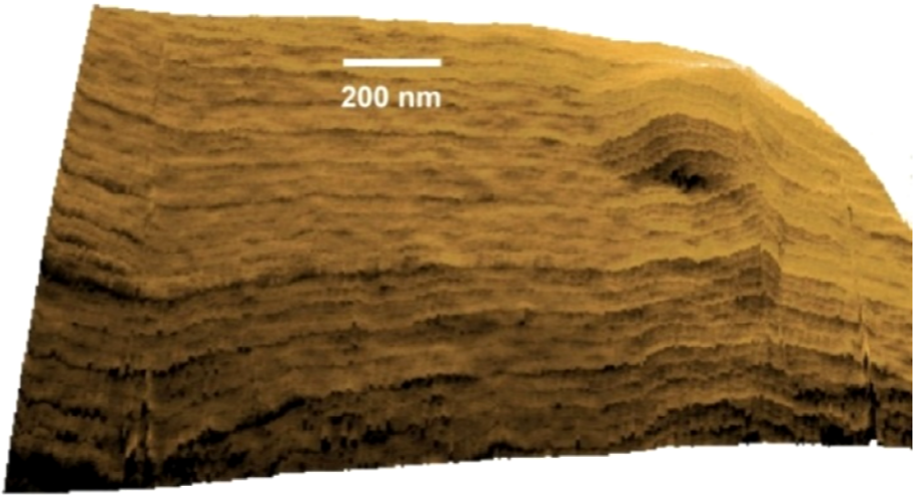
Tridimensional view of a confinement region (lateral wall) formed by a protrusion arrangement on the Nafion surface. The depth is ∼ 790 nm, and the extension is 2000 nm along the *X* and *Y* axes.

In order to further characterize the hydrated Nafion topography, a lateral profile of a trough was probed. Figure 5 shows the full view of another protrusion and the cavity in its central region.

**Figure 6:**
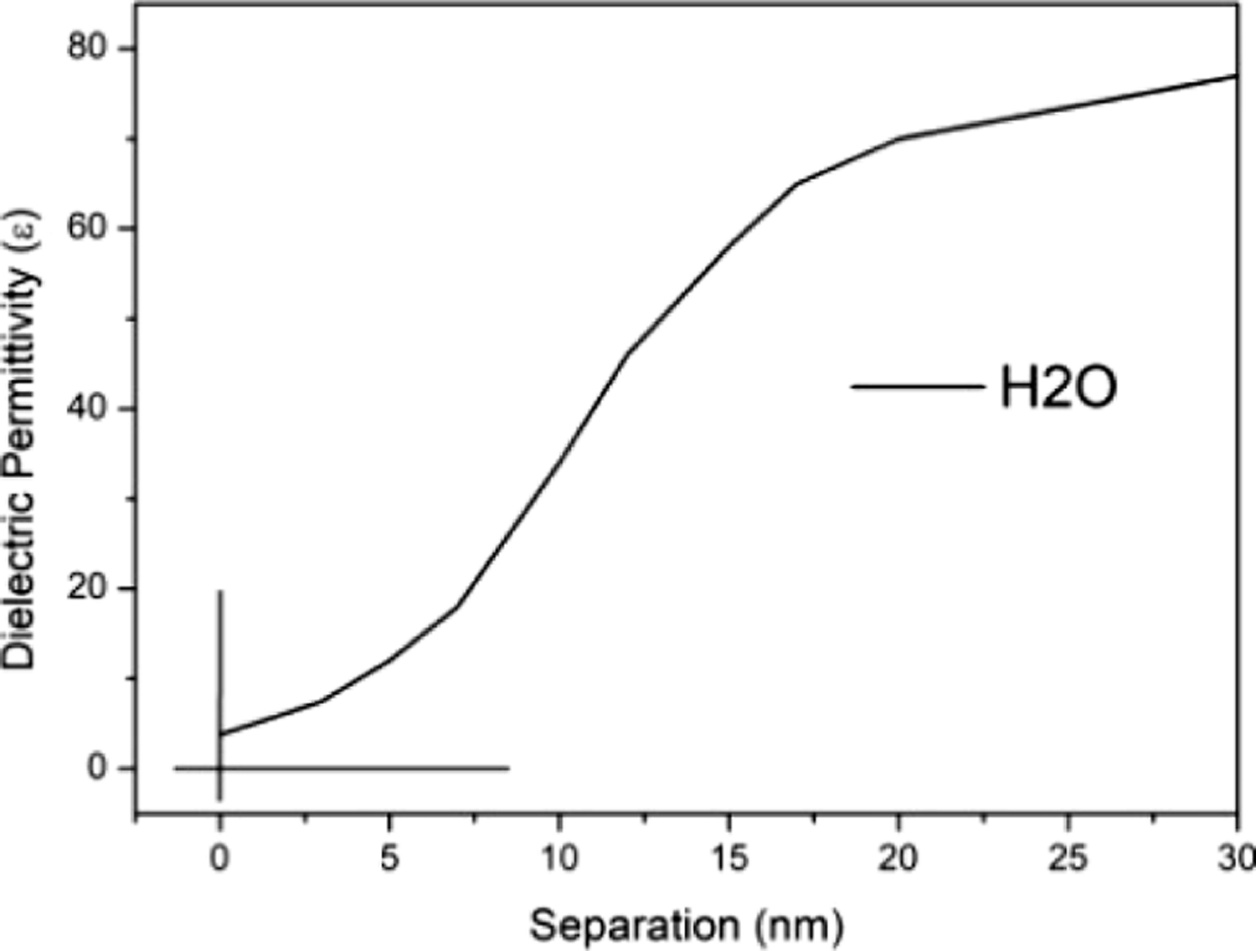
Interfacial water dielectric permittivity attached to mica.

After profile surface characterization, let us return to the force vs separation curves probing water interfacial regions. In the next section, we are going to describe the hydrophobic and hydrophilic character of these regions by determining the dielectric permittivity of the probed regions and compare these values to the dielectric permittivity of the interfacial region attached onto the hydrophobic and hydrophilic surfaces.

## 4 Discussion

### 4.1 Dielectric Permittivity of Interfacial Water Attached to Hydrophilic Substrates

The properties of liquid water in interfacial boundaries often exhibit notable anomalies. One of these anomalies is the variable dielectric permittivity profile; let us then describe the technique used to determine this profile.^15,25,32^

Patterns corresponding to the water interfacial region attached to hydrophilic surfaces (mica substrates) were previously published.^33^ The force is defined by eq 1 below. The electric displacement vector (*D*) is assumed to possess an exponential spatial dependence *D*(*z*) = *D*_0_e^–κ*z*^, and the vector amplitude (*D*_0_) is determined by the ionic charge distribution on the mica surface (*z* = 0) by using the Gauss law. The elemental volume (d*v*) of the trapezoidal tip immersed in the double-layer region is defined by d*v* = π[*R* + *z*·(tg α)]^2^ d*z*, where *z* denotes the integration variable of the trapezoidal volume and *x* denotes the distance between the surface and the end of the tip.

The electric energy variation involved in the exchange of the relative permittivity of the double layer with that of the tip is calculated by integrating the energy changes as a function of the tip distance from the substrate over the tip-immersed volume in the double layer region. The force is obtained based on the gradient of energy expression, i.e., *F*_*x*_ = −grad Δ*W*, where
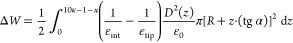
1

The measured force vs separation curves are adjusted to eq 1 using the interfacial dielectric permittivity as a parameter. The dielectric permittivity profile in the interfacial region for the hydrophilic mica substrate immersed in water is shown in Figure 6. For a mica substrate, ε_int_ = 4 is the value of the permittivity at the substrate, and the variable ε(*x*) extends ∼30 nm away from the surface.

For hydrophobic substrates,^32^ we have proposed that the polarization charge associated with the interfacial structure of broken hydrogen bonds generates an electric field that aligns the water molecules in the polarization layer.

As shown for hydrophobic surfaces, the size of the force acting on the tip is given by the energy change involved in the immersion of the tip inside the polarization layer; which is given by the product of the immersed tip volume times the dielectric permittivity variation and times the square of the electric field vector; observe that for hydrophilic substrate, the force is calculated using *D̅*. The tip was defined to have a sharpened conical shape with one flat end with an area of π*R*^2^. The force is obtained by the gradient of the energy expression, i.e., *F*_*z*_ = −(∂/∂_*z*_)Δ*W*, where
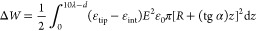
2

The polarization charge at the interface generates an electric field *E* with an exponential decay length *l*, i.e., *E*(*z*) = *E*_0_e^–*z*/*l*^, and the orientation of the water molecules is described by a spatially variable dielectric permittivity.^32^ The dielectric constant of the tip is ∼7, so the repulsive profile corresponds to the region with ε < 7.

The force vs separation curves shown in Figure 2a,b show some repetitive attraction and repulsive profiles, respectively. These repetitive profiles are going to be explained next.

### 4.2 Repetitive Step Profiles in the Force vs Separation Curves

The force vs separation profiles of Nafion interfacial regions shown in Figure 2a,b characterize the hydrophilic and hydrophobic regions, respectively. These water interfacial arrangements are associated with the Nafion surface spatial structure as will be explained next.

**Figure 7:**
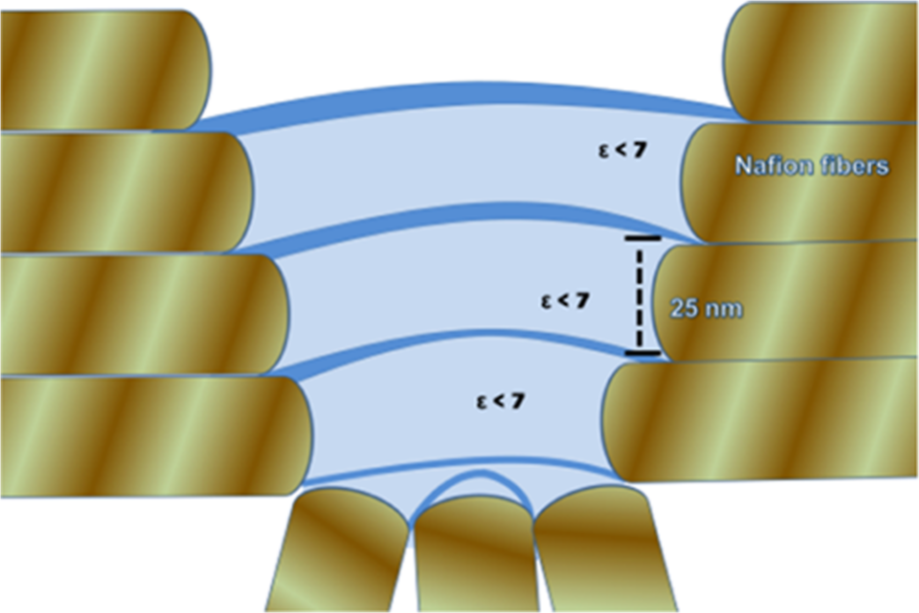
Schematic arrangement of the formed water interfacial arrangement at Nafion surface protrusions in hydrophilic domains. The lower part of this region is immersed in water.

Nafion interfacial hydrophilic sites (Figure 2a) show various attraction spikes in force vs separation profiles, which are associated with the region with ε < 7. Since the force vs separation profile in Figure 2a shows attractive spikes with the size of the dimension of the Nafion fibrillar diameter, we claim that this dimension is linked to the fibrillar structural dimension (∼25 nm) or to its formed ∼50 nm pair. It is then associated with the dielectric permittivity value ε < 7 present in these regions attached to fiber hydrophilic domains. The structure that generated these attraction regions in the force vs separation curves is schematically shown in Figure 7, where each fiber pair is linked to a water channel with ε < 7. These large numbers of steps are associated with the substrate topography, and the steps reflect the attached layer of the water structure as shown schematically in Figure 7.

**Figure 8:**
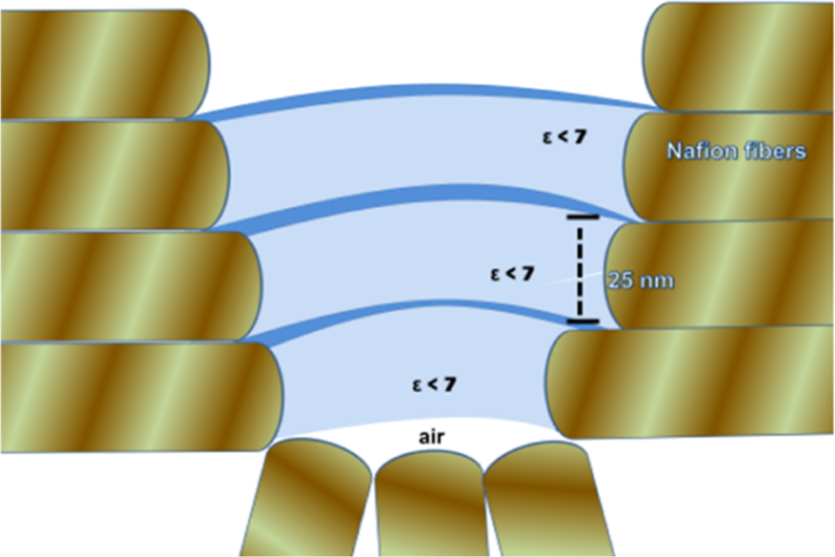
Schematic arrangement of the formed water interfacial arrangement at Nafion surface protrusions in hydrophobic domains, where a layer of air (vapor) is present at its bottom.

By probing these layers with ε ∼ 7 steps, we observed that they are present attached to hydrophobic surfaces too. The 25–50 nm step size dimensions shown in Figure 2a (hydrophilic section) are similar to the step dimensions present at the hydrophobic regions (Figure 2b). We then claim that this water arrangement generated at the hydrophilic sites extends to the hydrophobic site interfacial region generating the structure schematically indicated in Figure 8.

The Nafion surface topography formed by hydrophilic and hydrophobic regions when present in a confined region (Figures 4–6) is then responsible for this nanosized water arrangement. We have observed distinct surface structures at Nafion hydrophobic or hydrophilic sites which induce the formation of a channel that extends from hydrophilic to hydrophobic sites inside the surface protrusions.

We then claim that the observed interfacial water structure is formed by almost equal size ∼25 nm channels connected to the hydrophilic fibers, forming the Nafion surface confined in a protuberance. The Nafion surface hydrophilic and hydrophobic domains modify the dielectric properties in the interfacial region, forming a well-defined arrangement. These arrangements are formed between opposite hydrophilic lateral sites and they may extend to the hydrophobic regions as shown in Figure 8 in the form of channels. Our results possibly provide a useful microscopic picture of the water interfacial structure attached onto Nafion.

## 5 Conclusions

Attached to Nafion surface interfacial regions, repetitive attractive/repulsive profiles were observed in the force vs separation curves which corresponded to water molecular arrangements, i.e., water nanochannels formed by tubular hydrophilic domains. These nanodomains generated by the Nafion surface hydrophilic regions may extend to the hydrophobic regions. Water nanochannels were identified by measuring force vs separation interfacial profiles which are characterized by the region, where ε_r_ < 7 implies an arrangement of a very organized water structure when compared to bulk water with ε_r_ = ∼80. These arrangements are confined inside protrusion cavities present at the Nafion surface. In conclusion, nanosized water structures are then observed induced by nanosized Nafion surface spatially variable wettability.
